# Capturing the Biologic Onset of Inflammatory Bowel Diseases: Impact on Translational and Clinical Science

**DOI:** 10.3390/cells8060548

**Published:** 2019-06-06

**Authors:** Dario Sorrentino, Vu Q. Nguyen, Maithili V. Chitnavis

**Affiliations:** 1 IBD Center, Division of Gastroenterology, Virginia Tech Carilion School of Medicine, FRACP 3 Riverside Circle, Roanoke, VA 24016, USA; vqnguyen@carilionclinic.org (V.Q.N.); mvchitnavis@carilionclinic.org (M.V.C.); 2 Department of Clinical and Experimental Medical Sciences, University of Udine School of Medicine, 33100 Udine, Italy

**Keywords:** pre-clinical disease, Crohn’s disease, ulcerative colitis, biologic onset, disease markers, screening, microbiome, early diagnosis

## Abstract

While much progress has been made in the last two decades in the treatment and the management of inflammatory bowel diseases (IBD)—both ulcerative colitis (UC) and Crohn’s Disease (CD)—as of today these conditions are still diagnosed only after they have become symptomatic. This is a major drawback since by then the inflammatory process has often already caused considerable damage and the disease might have become partially or totally unresponsive to medical therapy. Late diagnosis in IBD is due to the lack of accurate, non-invasive indicators that would allow disease identification during the pre-clinical stage—as it is often done in many other medical conditions. Here, we will discuss what is known about the biologic onset and pre-clinical CD with an emphasis on studies conducted in patients’ first degree relatives. We will then review the possible strategies to diagnose IBD very early in time including screening, available disease markers and imaging, and the possible clinical implications of treating these conditions at or close to their biologic onset. Later, we will review the potential impact of conducting translational research in IBD during the pre-clinical stage, especially focusing on the role of the microbiome in disease etiology and pathogenesis. Finally, we will highlight possible future developments in the field and how they can impact IBD management and our scientific knowledge of these conditions.

## 1. Introduction

The incidence and prevalence of inflammatory bowel diseases [IBD]—Crohn’s disease [CD] and ulcerative colitis [UC]—is increasing steeply in Western countries and around the world, including Asia, a continent that not long ago seemed to have been spared by these conditions [1,2,3]. At this pace, the IBD burden in the world will soon take the shape of an epidemic of massive proportions. Much of the impact of these diseases is due to the fact that they are diagnosed late in time. When symptoms start it often takes several months on the average to diagnose UC and especially CD [4]. This is partly due to the complex nature of these diseases [5], to their clinical similarity to more benign, frequent conditions in the same age population (e.g., irritable bowel syndrome [IBS]) and to other still incompletely defined factors [4]. Even when IBD are promptly diagnosed at symptom onset a significant proportion of patients might already present with strictures and other complications [6,7,8]—this suggesting that the disease has already run a long silent course causing extensive damage. Hence, therapeutic response in IBD is often poor with patients often requiring hospital admission and surgery despite the great progress made with new medications. In addition to the negative impact on clinical outcomes, diagnosing IBD late in its course has also hampered our understanding of its causes and pathogenesis—and has significantly slowed research progress.

These considerations emphasize the importance of diagnosing IBD in the pre-clinical phase, aiming at preventing complications and possibly the clinical onset. Here we will especially focus on CD and describe what is known on its pre-clinical disease and natural history. Next, we will discuss whether IBD can be diagnosed at a very early stage by imaging or by non-invasive markers and whether it could be truly predicted before inflammation has taken place. We will then focus on the potential implications of conducting translational research in the very early disease stages—with an emphasis on the role of microbiome in IBD etiology and pathogenesis. Finally, we will discuss how this research and clinical field might develop in the near future and how it might advance our scientific knowledge and the clinical opportunities for patients with IBD.

## 2. What We Know about Preclinical CD

As of today, pre-clinical CD is a poorly known entity. It starts with the biologic onset of the disease and progresses over time until the appearance of symptoms. Little is known about the natural history of pre-clinical CD and much of what we know is based on its course after diagnosis. We know that the large majority of CD patients will develop complications (especially strictures and fistulas) over the long term if left untreated [6,9,10] due to the basic process of inflammation, repair and collagen deposition [6,7,8,9,10,11,12].

Since patients at diagnosis might already present with complications [4,6,7,8,13,14,15], it is clear that—in general—by the time the disease has caused symptoms it might have already run a long, several years-course. This conclusion is also based on the natural history of post-operative CD recurrence—which starts with minimal, asymptomatic inflammation at the neo-terminal ileum [16] and later proceeds to more severe inflammation, symptomatic relapse and stricturing. Indeed, it has been shown that the interval between resection, disease relapse and development of strictures requiring surgery is approximately 8–9 years [17,18,19,20]. Esch and colleagues have followed over time 43 patients with CD lesions found accidentally during endoscopy performed for standard of care indications or because of associated diseases (ankylosing spondylitis, primary sclerosing cholangitis and fissura-in-ano) [21]. During follow-up (median 78 months range 3–216), they found that 31 patients (72%) developed symptoms after a median time of 46 months (range 2–109) an evolution similar to that of a CD group operated on, with 5-year cumulative risk of symptomatic disease of 0.64 ± 0.08 [21]. Other studies [22,23] have focused on asymptomatic patients diagnosed by “chance” during colonoscopy for CRC screening and found that symptoms might develop relatively earlier after the index endoscopy. However, patients subjected to CRC screening are older than typical IBD patients and in that study already positive for fecal occult blood [23]—this indicating the presence of significant mucosal lesions and relatively advanced disease at diagnosis.

Undoubtedly, given the limitations of the diagnosis by “chance”, adequate information on CD natural history can only be gained by the prospective follow-up of individuals diagnosed at or near the disease biologic onset by dedicated CD screening.

However, screening the general population for CD would not be feasible since the yield would be low [24]. However—given the disease well known genetic component—screening family members of CD patients might guarantee a better yield [25].

We have conducted such a screening study with ileo-colonoscopy in a group of 38 first degree relatives (FDR) of CD patients [26] with the primary goal of identifying patients with very early, asymptomatic disease among individuals apparently healthy and in whom any additional cause for intestinal inflammation had been excluded. Tissue, blood and stool samples were collected for a number of tests.

**Figure 1:**
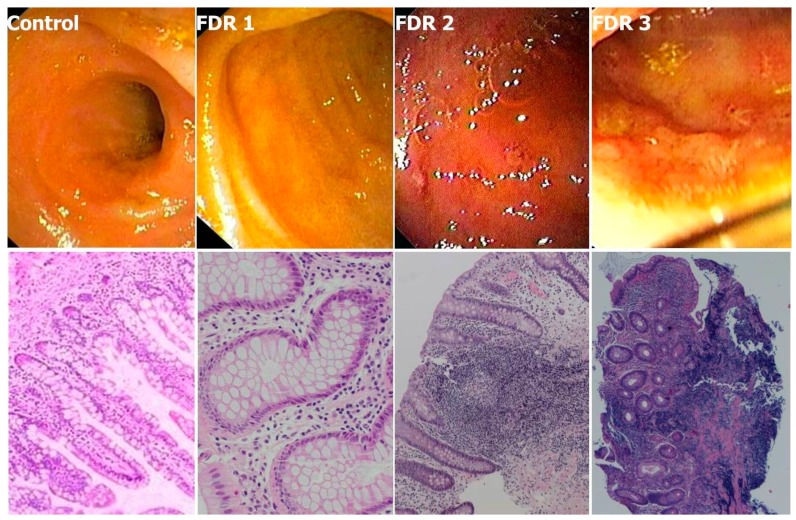
The three FDR phenotypes compared to controls in the colonoscopy based screening study. FDR1: normal—identical to controls; FDR2: with tiny aphthae or superficial erosions; and FDR3: with frank but limited and very early CD inflammation and ulcers. Corresponding phenotypes at histology were for FDR1: normal; for FDR2: mild inflammatory lesions including crypt reduction, chronic inflammatory infiltrate, lymphoid hyperplasia and superficial erosions; and for FDR3: histological features typical of CD with more severe inflammation, crypt distortion, focal fibrosis of muscularis mucosae, and superficial ulcerations (H&E, 10✕ except FDR1: 20✕). Reprinted with permission from [26].

An age-matched control group consisted of individuals who had tested completely negative at colonoscopy and other tests performed for clinical indications (e.g., rectal bleeding, IBS). We identified three individual FDR phenotypes (Figure 1): (1) normal—identical to controls (FDR1); (2) with tiny aphthae or superficial erosions (FDR2); and (3) with frank but limited and very early CD inflammation (FDR3). Lesions, when present, occurred at the ileocecal valve and/or the terminal ileum. At histology the corresponding phenotypes were (Figure 1): (1) normal; (2) mild inflammatory lesions including crypt reduction, chronic inflammatory infiltrate, lymphoid hyperplasia and superficial erosions; and (3) histological features typical of CD.

Histology scoring and Divisive Clustering Analysis (DIANA) produced three highly separated clusters as above with divisive coefficient D = 0.94. Overall, 10% of FDR had frank CD at endoscopy and histology (FDR3) whereas 30% of individuals had minimal, non-evolving inflammation (FDR2). Hence, 40% of all FDR had inflammation—a proportion very similar to that defined as bearing “subclinical inflammation” and identified by elevated fecal calprotectin (FC) levels in FDR by Thjodleifsson et al. [27] (see section below). Therefore, the group of patients with subclinical inflammation in Thjodleifsson’s study appears to be formed by individuals with endoscopic and histological features of CD (who might develop the typical clinical picture over time) and by those with minimal inflammation (who do not appear to develop symptoms in time).

**Figure 2:**
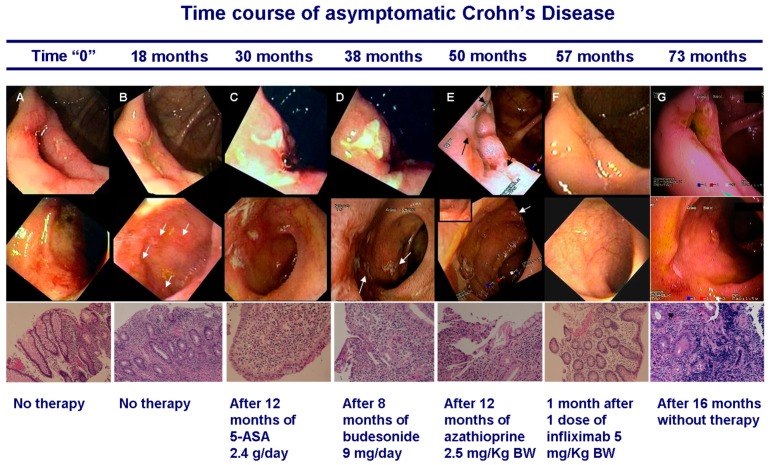
Time course of pre-clinical Crohn’s disease. Ileo-cecal valve and terminal ileum images at endoscopy and ileo-cecal valve histology at different times during the patient follow-up. Images taken at the identical location. (**A**) Diagnosis (Time “0”). Endoscopy: small superficial ulcerations/erosions. Histology: focal inflammation, microaphtoid lesions and microhemorragies. H&E 10✕. (**B**) At 18 months—no treatment. Endoscopy: new superficial ulcerations in the valve and terminal ileum (arrows). Histology: worsening of the inflammatory infiltrate with extensive erosions; vascular congestion and “summit lesions”. H&E 10X. (**C**) At 30 months—after 12 months of mesalamine 2.4 **g/day.** Endoscopy: larger and deeper ulcerations in the valve; new ulcerations in the terminal ileum. Histology: marked, diffuse inflammatory infiltrate. H&E 10X. (**D**) At 38 months—after 8 months of budesonide 9 mg/day. Endoscopy: larger valve ulcerations; new ulcerations more proximally in the terminal ileum (arrows). Histology: erosions and aphtoid ulcers with disruption of mucosal epithelial superficial and cryptic lining. H&E 10X. (**E**) At 50 months—after 1 year of azathioprine 2.5 mg/Kg. Endoscopy: more confluent and superficial valve and terminal ileum ulcerations (black arrows); new small ulcerations appear more proximally in the terminal ileum (white arrow and inset). Histology: persistent erosions and aphtoid ulcers with partial preservation of mucus production. H&E 10X. (**F**) At 57 months—6 months after stopping azathioprine and 1 month after 1 infliximab infusion 5 mg/Kg. Endoscopy: minimal residual inflammation in the valve; normal terminal ileum mucosa. Histology: mild edema and slight inflammation, with preserved epithelial architecture and mucin production. H&E 10X. (**G**) At 73 months—after 16 months without therapy. Endoscopy: deep valve and terminal ileum ulcerations. Histology: severe inflammation, erosions and lymphoid focuses. H&E 10X. Reprinted with permission from [28].

Among the FDR3, we followed for several years the 27 year old sister of a CD patient [28]. The initial colonoscopy and histology (Figure 2A) showed hyperemia and small superficial ulcerations/erosions in the ileo-cecal valve and the terminal ileum with histology showing focal inflammation in the lamina propria, shallow microaphthae and hystioid aggregates. It was initially decided to observe the disease evolution by colonoscopy. After 18 months endoscopy and histology worsened (Figure 2B) and we initiated therapy with the goal of blocking disease evolution. However, standard doses of mesalamine and budesonide—given for 12 and 8 months respectively—did not impact on disease progression (Figure 2C,D). Azathioprine 2.5 mg/Kg was then given for 12 months. While endoscopy and histology showed a modest to moderate improvement several new small ulcerations appeared more proximally in the ileum (Figure 2E). Due to the patient’s concern about disease progression and after extensive discussions one single infusion of infliximab (5 mg/Kg) was attempted after azathioprine wash-out. Several weeks after the infusion ileocolonoscopy and histology only showed a minimal degree of residual inflammation (Figure 2F). At this point the patient elected to stop all therapies—returning for colonoscopy 16 months later (>6 years after the initial diagnosis) still completely asymptomatic. Colonoscopy and histology showed disease recurrence and severe progression with deep ulcerations (Figure 2G), lymphoid follicular hyperplasia and focal fibrosis in the lamina propria and submucosa. The patient has been subsequently treated per CD standard of care after the disease clinical onset—more than 9 years after the initial diagnosis. 

To our knowledge, this is the first reported case of prospectively studied pre-clinical CD. This study shows that diagnosis of pre-clinical CD is feasible by screening asymptomatic FDR and that early diagnosis can potentially lead to improved outcomes and perhaps even allowing to halt disease progression. Importantly, the time between diagnosis and significant tissue damage/symptom onset appears to be long enough to afford diagnostic and interventional studies. 

## 3. Is Screening for IBD Feasible? In Whom? And How Should it be Done? 

Diagnosing IBD at the biologic onset can only be done by screening. Since screening the general population is unfeasible because of the low yield the only other approach would be screening individuals at risk for the disease as well as discovering non-invasive markers that could lead to imaging and to a definite diagnosis.

Thjoldefsson et al. have conducted almost two decades ago one of the most interesting screening studies in FDR [27]. In general, clustering studies have shown that 5–15% of patients have a family history of CD [29]. However, these authors showed that ≥40% of FDR have intestinal inflammation as evinced by elevated FC levels, a sensitive marker of intestinal inflammation—with values intermediate between those of healthy controls and their affected relatives [27]. This interesting observation, confirmed by other studies [30] and also reported in UC [31], had never received a proper explanation and has intrigued the scientific IBD community for a long time [32]. The authors of the original study interpreted those findings with a genetic model of additive inheritance (causing a disease phenotype in FDR in between normal and affected individuals). However endoscopy was not performed in that study (or in subsequent studies) [27,30,31]. In our own study [26] we confirmed and explained those findings since 10% of our FDR had frank CD at endoscopy and histology and 30% of them had minimal inflammation. Overall, 40% of all FDR had inflammation—a figure consistent with the proportion of patients with elevated FC levels reported by Thjodleifsson et al. [27]. A long follow–up (53 months on the average) showed that in individuals with minimal inflammation (FDR2) histology does not change over time. 

Other studies are also consistent with the existence of a third CD phenotype. Zhulina and colleagues [33] have shown activation of the NF-κB pathway and increased neutrophil activity (as measured by myeloperoxidase [MPO]) in intestinal tissues of a large proportion of discordant twin siblings of CD and UC patients when compared to healthy controls—this providing additional evidence that many “healthy” FDR of IBD patients bear a subclinical inflammation. Confirmation of such intermediate CD phenotype also comes from the finding that gut permeability is altered in both monozygotic twins discordant for CD [34]. Since only one of the twins developed the disease the authors of that study concluded that such defect is genetically determined and that environmental factors are crucial to trigger the disease [34,35]. Globally, these data indicate that FDR include a third phenotype, in between normal and affected individuals as originally postulated by Thjoldefsson et al. [27]. In theory FDR2 might bear some, but not all, the defect(s) that are both necessary and sufficient to cause the full blown disease. They might also bear differences that protect them from developing the disease [36]. Or, they might have simply avoided early exposure to a triggering environmental stimulus, the “final hit” leading to CD [37]. Likely, understanding why these individuals with the intermediate phenotype do not develop the disease might be key to comprehend the origin of the disease itself. For the time being, these studies highlight the complexity and heterogeneity of FDR phenotypes and the need to stratify FDR data for the different clusters for proper interpretation. So far most studies in this population have failed to recognize such heterogeneity.

Marker discovery studies have targeted IBD patients (army recruits, nurses, individuals followed for different indications such as cancer development) whose biologic samples were taken before disease diagnosis [38,39,40]. Markers tested have included anti-microbial antibodies (such as anti-Saccharomyces cerevisiae antibody [ASCA] IgA, ASCA IgG, anti-OmpC, anti-Fla2, anti-FlaX and anti-CBir1) auto-antibodies (perinuclear anti-neutrophil cytoplasmic antibodies [pANCA]) and markers of inflammation (such as highly sensitive-CRP, intestinal permeability, IL-6. The calculated relative risk (compared to matched controls) of those testing positive have been used to estimate the accuracy of such markers to diagnose the disease at the preclinical stage. The time from the first positive test to the disease symptomatic onset has been deemed to reflect the entire pre-clinical natural history. The success of such approach has been mixed. One of the best and most recent of such studies [40] has shown that 65% of CD patients were positive for at least one of six CD-associated anti-microbial antibodies in the earliest available sample, at a median of 6 years before CD diagnosis with the number of positive anti-microbial antibodies increasing up to the time of diagnosis. 

An important issue with the marker discovery studies is that - due to the absence of reliable and objective data (endoscopy, histology)—the disease stage at time of sample collection is unknown. Because of the lack of symptoms it is generally assumed that the disease was at a very early stage at the time of the first positive sample. However the lack of symptoms cannot be relied upon, since the disease can remain clinically silent for many years [28]. This fundamental aspect questions the true predicting power of such tests in diagnosing very early disease. Future studies will have to test potential markers in a prospective, interventional study whereby positive individuals are subjected to standard clinical tests (colonoscopy, imaging) *at time of sample collection*. 

A different marker discovery approach is to prospectively follow individuals at risk of developing the disease until they actually do. One such study uses a genetic, environmental and microbiome (GEM) cohort [41] and enrolls FDR aged 6–35 years of affected CD probands, prospectively collecting biological samples (blood, urine and stool), as well as environmental exposure. Evaluation in changes of biomarkers and exposures among those who develop disease compared with siblings who do not develop it at the end of the follow-up period might shed light on many aspects of preclinical disease and identify markers which could be used for general screening in the future. However, limited sample collection (at enrollment and at disease onset/end of the study) and the possible presence of the disease already at enrollment may reduce the practical impact of this study. Again, the absence of symptoms cannot be considered equivalent to disease absence in this context.

Overall, the possibility of diagnosing pre-clinical CD or predict its onset in asymptomatic individuals using ad-hoc non-invasive markers remains elusive at the moment [42]. 

Blood markers of inflammation such as CRP could in theory be positive in early disease. However, they are usually elevated only when the inflammatory burden becomes significant and might not be useful in this context, with the possible exception of highly sensitive-CRP which seems to be more sensitive than other blood markers of inflammation [43]. 

The fecal markers of intestinal inflammation (lactoferrin and FC) might be more sensitive and accurate than blood markers but their role in screening individuals at risk for IBD remains to be studied. Several [27,44,45] but not all studies [33] have shown that a large proportion of asymptomatic FDR of CD patients have elevated FC levels (see above). In our own screening study the FDR group had greater median values for FC (*p* < 0.02) compared to the controls [26]. Furthermore, FC levels appeared to correlate with endoscopy scores in one of the patients diagnosed with preclinical CD during follow–up and treatment [28]. However, only three out of four of the FDR diagnosed with frank CD (FDR1 group) had FC values above the normal threshold. More data are needed to evaluate the accuracy of these markers for initial screening in individuals at risk of developing IBD. Likely—like the blood markers – the fecal markers might become significantly elevated only when inflammation has caused significant damage and loss of tissue in the intestinal mucosa. In addition, they might be less sensitive when the inflammation is exclusively localized in the small bowel [46]. 

**Figure 3:**
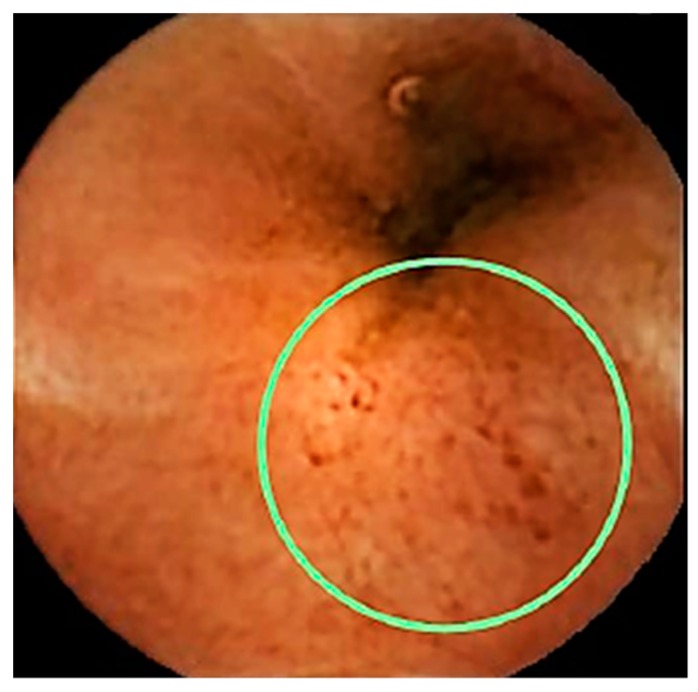
Capsule endoscopy image of a FDR enrolled in the screening study [49]. The circle shows an area of inflammation and small ulcers in the ileum.

Can imaging studies diagnose pre-clinical IBD? We have shown that ileo-colonoscopy can be used for the purpose [26] and would certainly be the most accurate test, being already the gold standard to diagnose both *clinical* UC and CD [5]. In practical terms it would be way too costly and invasive for colonoscopy to be used to routinely diagnose pre-clinical disease. In addition, it would not be diagnostic if the disease is localized proximally to the reach of the colonoscope. A test that might combine accuracy with low invasiveness—hence with great potential to diagnose pre-clinical CD—is capsule endoscopy. In a recent meta-analysis Park and colleagues [47] have shown that capsule endoscopy in patients with known or suspect CD has a higher yield than colonoscopy, push enteroscopy, barium studies and CT enterography and similar diagnostic accuracy of MR enterography—the current gold standard to diagnose small bowel CD. Teshima et al. [48] have tested this technique in asymptomatic relatives of CD patients and found it accurate to diagnose small bowel ulcerations [48]—but they did not verify findings by other means. In addition, capsule endoscopy has not been used—so far—to visualize both small bowel and colon for screening purposes in a FDR population. We are currently expanding our original study in CD-FDR using the new panenteric (“Crohn’s”) capsule endoscopy which allows visualization of the entire digestive tract, this allowing a one-off testing screening strategy [49]. In our trial, patients positive at capsule endoscopy will undergo colonoscopy to confirm findings and acquire tissue for histology. Additional tests will be performed in tissues, stool and blood samples to identify potential markers of early disease activity that could be potentially applied in the future to the general population. The current experience suggests that the Crohn’s capsule endoscopy is in principle an excellent, safe and relatively inexpensive mean to diagnose early disease in at-risk individuals (Figure 3). 

## 4. Can IBD Be “Intercepted” or “Predicted”?

Ideally, rather than aiming at diagnosing early disease, we should aim at intercepting IBD at a very early, pre-inflammatory biologic stage—and possibly block progression leading to inflammation. Clues as to whether this might be possible in IBD—especially CD—come from earlier studies in rheumatoid arthritis (RA), a chronic immune mediated disease that shares a number of features with CD [50]. In RA, regulatory T cells (CD4+ CD25+ FoxP3+ [Tregs]) seem to play a crucial role. They regulate the transition from acute inflammation to chronic inflammation as well as maintaining peripheral tolerance [50]. Inducing sustained drug-free remission in RA might be possible in patients with low inflammatory cell number, high Treg cell frequency and high proportions of naïve CD4+ T cells [51,52]. In RA, once remission has been induced, recurrence of disease activity takes place when effector T cells re-infiltrate the synovium [53]. Synovial recruitment of T cells is driven by selective homing, local homeostatic forces, or antigenic stimulation. To induce profound, long lasting remission the synovial T cells must be completely eradicated within the first few months of disease [53]. The short window of opportunity to normalize the immune system seems to be due to the inability of the thymus to control immunological homeostasis once inflammation or another unknown event has taken place and has profoundly affected the immune system. Once the window of opportunity closes the immunological status of the patient may then switch from reversible to controllable [54]. This model is also supported by the clinical data related to stem cell transplantation induced remission after immunological ablation [55]. Those data show that patients reaching long term stabilization have a peak in CD25-FoxP3 expression in CD4+ cells which have gained again their suppressive activity and are anergic to nonspecific stimulation—hence, the establishment of a renewed immune balance [55]. 

In CD, intestinal inflammation seems to result from an innate dysregulated expression of type 1 T-helper lymphocyte [Th1] and Th17-related cytokines followed by a dominant Th1 and Th17 adaptive response [56,57,58]. Resistance to effector T cell apoptosis leading to expanded Th1 and Th17 populations and continuous exposure to luminal antigens and adjuvants (which trigger their response) steer the inflammatory process towards chronicity [57,58]. 

The therapeutic efficacy of anti-TNF agents in CD can be in part explained by their ability to induce apoptosis of activated cells expressing TNFα in their membrane which—together with induction of cytotoxicity—results in a reduced number of inflammatory cells relatively to Tregs [59,60,61,62]. Moreover, TNF-blocking agents largely restore gut barrier function in CD [63] and affect many other inflammation related aspects [61,64,65]. 

**Figure 4:**
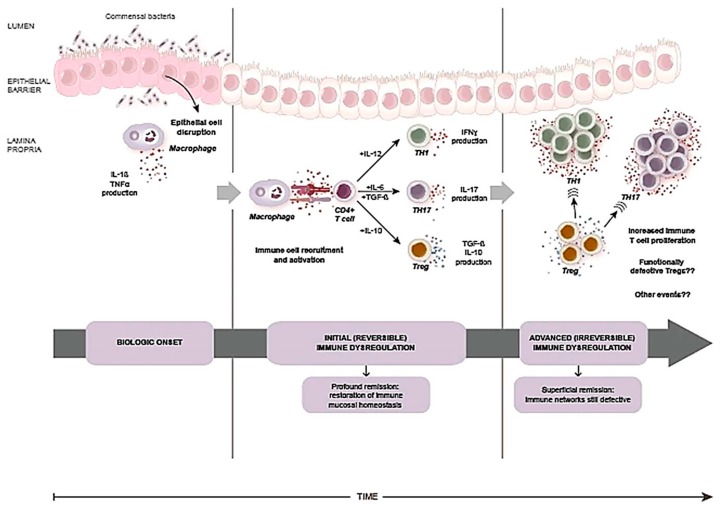
Biologic evolution and immunological events in Crohn’s disease. Unidentified environmental triggers might cause epithelial cell disruption and activate the innate immune system which leads to disease onset with cytokine production by macrophages and other cells. Immune cell recruitment and activation follows. It is possible that during these early disease stages dysfunctional immunoregulatory networks could be deeply restored by therapy. As the disease progresses mucosal homeostasis might become irreversibly altered. Initiating therapy at this stage might lead to superficial and transient disease remission and therapy must be continued. (Modified and reprinted with permission from Sorrentino D, et al. Stopping anti-TNF agents in patients with Crohn’s disease in remission: is it a feasible long-term strategy? Inflamm. Bowel Dis. 2014, 20, 757–766.

As in RA, use of anti-TNF treatment is highly effective in early, rather than long standing, CD [66]. This is likely the result of increased apoptosis of activated cells and induction of cytotoxicity which results in a reduced number of inflammatory cells relatively to Tregs [59,60,61,62]. This in turn favors the re-establishment of a balanced regulatory versus effector immune response, a state characterized by tolerance or controlled inflammation [62,67]. As in RA—once early treatment in CD has effectively down-regulated inflammatory tissue-damaging responses and restored mucosal immune homeostasis—the discontinuation of therapy could result in indefinite maintenance of remission [68]. By contrast, initiating treatment later in time can be effective but will need to be continued indefinitely (Figure 4) when immunoregulatory networks remain defective and have not been completely restored by therapy. 

Supposing it is indeed possible to block disease evolution—and essentially cure it—the real issue becomes diagnosing IBD at such pre-inflammatory stage so as to restore the mucosal immune homeostasis before the process becomes irreversible. The problem is twofold: first, identifying an accurate marker of disease activation in the absence of inflammation. Second, being able to take full advantage of a potentially very short therapeutic window of opportunity. At the moment, these are very daunting tasks.

Perhaps, a better objective would be to predict the disease—i.e., anticipating the disease process before it actually takes place. Can it be done? Current knowledge holds that a disease can be predicted only when it is genetically transmitted e.g., cystic fibrosis or Huntington’s disease. But this is not the case for IBD.

In IBD, monozygotic and dizygotic twin pairs have a concordance rate of <30% thus indicating that only approximately 1/3 of the risk of developing these conditions is genetic [69,70]. Initial linkage-based investigations focused especially on CD and on the NOD2 gene where a significant disease susceptibility locus was identified on chromosome 16 [71]. Later, Genome Wide Association Studies [GWAS] and other techniques identified >140 susceptibility loci [72] with the large majority of them harbouring non-coding variants [70]. Overall, the genetic risk prediction for CD (i.e., the risk of developing the disease) appears of little clinical utility with current calculated chances of 12% to harbour/develop the disease for individuals testing positive to all the genetic tests [70].

**Figure 5:**
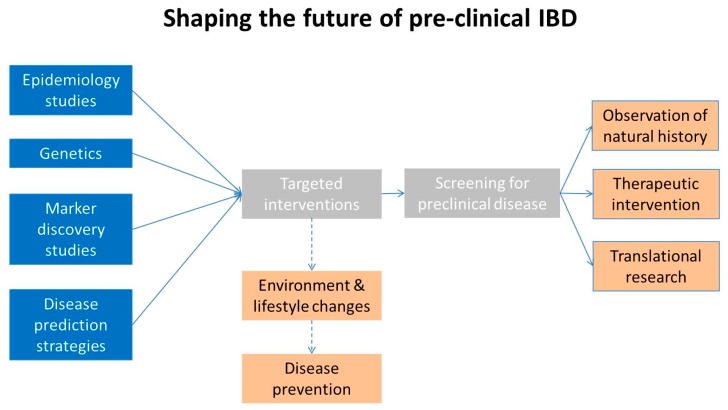
The future state of pre-clinical IBD. Ongoing epidemiology and marker discovery studies as well known genetic alterations and disease prediction strategies could lead in the future to specific intervention to reduce or eliminate environmental factors and eventually to disease prevention. In the foreseeable future they might allow the selection of individuals at risk for developing the disease who can be subjected to screening by imaging. Once a population with biologic onset-IBD is identified observational, therapeutic and translational studies could be performed.

Hence it is clear that genetics alone cannot predict IBD—at least based on currently available data. However, the genetic risk together with information derived from epidemiology studies could be used for potential lifestyle changes and disease prevention (Figure 5). Such information, together with the availability of disease markers, could be combined into disease prediction strategies, an approach that has been successfully attempted for a number of conditions using data mining and risk factors [73], artificial intelligence and neural networks [74] and other methods which use demographic, behavioral, geographic and medical variables. Conceivably, such tools could be used in the future to target for screening selected individuals at high risk of developing IBD. Identification of the disease at biologic onset will be instrumental to describe its natural history, intervene on disease evolution and possibly discover its causes (Figure 5).

## 5. What Is the Potential Impact of Translational Research in Preclinical IBD? The Example of the Microbiome

Both established CD and UC are known to be associated with a number of alterations in the intestinal structure and function. Such alterations have traditionally been the focus of clinical and basic research aimed at identifying the cause(s) and pathogenesis of IBD—and possibly target them for therapeutic purposes. However the unsolved issue with most of these alterations is whether they are true etiologic factors or are simply the result of long standing inflammation. This is a major question as it relates to disease understanding and treatment—which could be potentially clarified by studying the disease at its *documented* biologic onset. For example, intestinal barrier function is considered a crucial pathogenetic factor in IBD, invariably present in established disease [75]. Past studies have shown altered intestinal permeability in FDR [76]—this observation suggesting that intestinal permeability might be at the basis of IBD [77]. However increased intestinal permeability does not seem to be strongly associated with small bowel ulcerations at capsule endoscopy in FDR [48]. Likewise, inflammation in established IBD seems to be driven by well known proinflammatory cytokines such as TNF-a, IL-1, IL-21, IL-23 but also by IL-17 as well as by a decreased number of Treg cells [75]. Yet, preliminary studies in patients diagnosed with preclinical CD [28] reveal a surprizing negative correlation of inflammation with IL-17 and a positive correlation with the anti-inflammatory cytokine IL-10 and with Treg cell number, findings potentially consistent with tissue resistance to Tregs’ activity [57] or ineffective blockade of Th1 response, a potential driver of inflammation at least in CD [78]. Similarly, although elevated in both established disease and FDR, the pathogenetic meaning of positive serological antimicrobial markers [79,80,81,82] and of alterations in markers of innate and acquired immunity [33,83] remains elusive—and could be clarified in accurately diagnosed preclinical CD. 

Perhaps the best example that epitomizes the cause vs. consequence dilemma of the many reported alterations in established IBD is the disruption of the intestinal microbial profile—a subject that has attracted considerable research and clinical interest in the past several years. The rest of this section will focus on this aspect. 

The term microbiome refers to the collection of genes in an ecological community of microbes which include bacteria, archae, fungi, viruses, and phages. The human gut harbors on average 10^14^ microbes, or up to 10 microbes for every human cell. The human gut microbiota consists predominantly of bacterial microbes, in particular *Bacteroidetes* and *Firmicutes* which account for over 90% of the gut microbiome [84]. They appear to act as a digestive organ, a training tool for the immune system, and to protect us against infections.

Several studies have consistently demonstrated the presence of dysbiosis—an altered microbial profile—in patients with IBD. Ott et al. demonstrated that compared to controls, microbiome diversity was reduced by 30–50% in active IBD [85]. The lack of biodiversity in IBD is largely due to lower abundance of the phylum *Firmicutes*. Other typical alterations found in IBD patients compared to healthy controls include a decrease in *Bacteroides, Clostridia, Ruminococcaceae, Bifidobacterium, Lactobacillus, and F. prausnitzii* species; an increase in *Gammaproteobacteria*; and the presence of adherent invasive *E.coli* and *Fusobacterium*. Altered microbial function, metabolism and membrane transport has also been reported in IBD patients [36]. Because of these alterations in established IBD - and given the complex and vital role of bacteria in numerous physiological functions—it is presumed that the disruption of the “normal” microbial profile could be at the origin of these diseases. 

The ability to analyze and manipulate the gut microbiome also allows for potentially novel diagnostic targets in IBD. For example, the pretreatment microbiome composition has been found in several human studies to differ significantly between IBD responders versus non-responders. Ananthakrisnan et al. showed that community diversity was significantly higher with relatively greater abundance of *Roseburia inulinivorans* and *Burkholderiales* species at baseline among CD patients who later achieved remission with vedolizumab [86]. Using pretreatment microbiome data and statistical computation, Shaw et al. were able to predict treatment response with high accuracy [87]. Another possible diagnostic use of the altered microbial profile in IBD has been recently proposed by Choung et al. [40] whereby microbial related antibodies appear to be able to predict IBD in asymptomatic patients. Therapeutics based on microbiome restoration are also being developed—with the hope of impacting disease evolution [88]. Studies are also exploring the potential impact of the transmission of the microbial profile by IBD pregnant women to their babies and its possible therapeutic manipulation at birth [89].

However, as with the other reported alterations, whether dysbiosis is truly a causal factor or a simple consequence of IBD—i.e., bacterial adaptation for survival—is not entirely clear yet. It appears that genetic and environmental stimuli might affect the composition of the intestinal microbiota which in turn might play a crucial role in IBD pathogenesis due to its interaction with the mucosal immune system [36,90].

Current evidence might be consistent with both views. A number of findings suggest a potential etiologic role of the microbiome in IBD. For example, one bacteria subgroup *F. prausnitzii*—believed to be involved in modulation of inflammation and maintenance of the gut epithelial barrier—has consistently been found in lower quantity in fecal samples of patients with active IBD compared to those with inactive disease and controls [91,92,93]. On the other hand, pro-inflammatory Proteobacteria species—such as *Escherichia coli*—that can adhere and disrupt intestinal barriers have been found in overabundance in patients with CD [94,95]. In addition, dysbiosis seems to be associated with genetic polymorphisms. As discussed above, genetic studies and GWAS have identified multiple IBD susceptibility loci that are involved in host microbial interactions [72]. NOD2—the first susceptibility gene identified for CD [71,96]—is involved in intracellular bacterial sensing and modulation of the innate immune response to foreign pathogens via the NF-kB pathway. Individuals with mutations in two NOD2 alleles carry 30–40 times higher risk of developing CD. NOD2 mutations have been found to be associated with changes in microbial composition in ileal biopsies of CD patients, suggesting an interdependence relationship between genetics and gut microbiome in CD pathogenesis [97]. Another well known susceptibility gene—ATG16L1—is involved in the autophagy pathway that protects against colitis. Dendritic cells of ATG16L1-deficient mice do not induce regulatory T cells to suppress mucosal inflammation in response to *Bacteroides* protein secretions [98]. Finally, one of the best arguments for an important role of the microbiome in IBD pathogenesis is the impact of fecal microbial transplant (FMT) on IBD. 

FMT was first reported to be effective thirty years ago in a patient with UC [99] who had active disease for 11 years but was able to achieve symptomatic remission after instillation of a large-volume retention enema from a donor who was deemed to have normal colonic flora. More recent studies [100,101] have evaluated the impact of FMT in UC over a six to eight week treatment period, and found significant differences in clinical remission and (less often) in endoscopic response between those who received FMT compared to those who received placebo. In metanalyses the benefit of FMT has been found to be 28–33% over placebo [102,103]. In some of these studies a partial restoration of the microbial profile was reported in those responding clinically [100]. As expected, patients treated with pooled donor stool FMT do better than those treated with autologous FMT [104] and achieve a greater microbial diversity. No clinical trials on CD patients have been performed. In one metanalysis, the pooled proportion of patients from six cohort studies with CD who were treated with FMT achieving clinical response was 52% [102]. Unfortunately many of these studies cannot be compared or even be evaluated for true efficacy. In fact, in most cases the routes of administration are different and most often patients are also given 5-ASA, immunomodulators and biologics—some with a known delayed onset of action—which could have actually been responsible for the improved clinical outcomes in those treated with FMT [105]. Few studies have systematically used endoscopy, biochemical markers and microbial profile as primary study outcomes. Finally, one single treatment of FMT does not appear enough to achieve remission in IBD [106] as it is often the case for the treatment of *Clostridioides difficile* infection. Hence, for the time being it is unclear whether there is a true cause-effect relationship between FMT, the microbial profile and the clinical outcomes. Improvement seems to take place in one quarter to one third of patients—in most cases treated with other medications—this suggesting that clinical response might be associated with specific bacteria and metabolic pathways in both donors and patients. A recent RCT, conducted in a number of centers in Australia, seems to be consistent with such hypothesis [107].

Other observations seem to be consistent with the lack of impact of the microbiome in IBD etiology and pathogenesis. For example, the degree of inflammation impacts on the microbial profile in IBD and effective IBD medications (such as anti-TNF-α agents) seem to restore at least in part many of the microbial defects in IBD patients [108]. In addition, a dysbiosis similar to that described in IBD has been observed in patients with nonspecific intestinal damage [90]. Furthermore, it is known that identical twins discordant for CD have essentially superimposable microbial profiles [109] and it is clear that IBD cannot be treated by antibiotics or probiotics [110]. Moreover, the microbial profile is affected by a number of independent factors (both in health and disease) including age, diet (including the presence of food additives) smoking and perhaps ethnic origin [90]. In fact, dysbiosis and altered microbial metabolism is often present in healthy spouses of IBD patients—this finding clearly implying that the microbiome might be partially transferred from patients to healthy individuals. Indeed, cohabitation is a way to share our microbial cell population with family members, or even with our dogs [111]. However, spouses of IBD patients (or their dogs!) do not have an increased risk of developing the disease [111]. These observations suggest that local changes in the intestinal mucosa *following* the disease onset might be the cause of the altered microbial profile in IBD.

There are clearly limitations in understanding microbiome research both at the molecular and at the clinical level. Differences in microbial profile and function might be difficult to identify because of the inaccuracy of the sequencing methods, because protein synthesis in bacteria has unique features—different from those of mammals’; because some elements of the microbial community might be very scarce and hence difficult to compare among different groups (and yet they could play a vital role) and because of many other confounding factors. 

Regardess of the current evidence for and against an etiologic role of the microbiome and the other structural and functional abnormalities in IBD the greatest obstacle in understanding their role in IBD is the very long time gap between disease onset and established disease—as we diagnose it today. Indeed, the dysbiosis in IBD patients might not have been present several years earlier during the preclinical course, in the absence of substantial inflammation. And yet, the interest in this entire field has been triggered by the observation of the altered microbial profile in established disease compared to healthy subjects. This is why diagnosing IBD at the preclinical stage can provide important information to solve this and many other scientific dilemmas. Using ileo-cecal tissue DNA from biopsy samples collected during our initial FDR screening study [26] we employed next generation sequencing 16S (bacterial) for microbial analysis in the three FDR groups, healthy controls and in patients with active untreated established CD [112]. We also inferred the metagenomic functions of 41 microbiome-related cell processes by Phylogenetic Investigation of Communities by Reconstruction of Unobserved States analysis with metagenomic prediction based on the Kyoto Encyclopaedia of Genes and Genomes. When compared to healthy controls, as expected [86] microbial diversity in CD patients was decreased. However, in FDR—as a group and as individual subgroups—the diversity profile was not decreased compared to healthy controls. In Principal Coordinates Analysis (PCoA), ellipse centroids of FDR were positioned in opposite quadrants compared to CD patients, but close to the centroid for healthy controls. Inferred metagenomics pathway analysis for the five groups showed similar features. Thus, while intestinal microbial profiling confirmed significant dysbiosis in CD patients compared to healthy controls, FDR—including FDR3 (i.e., CD at its biologic onset)—showed a microbial profile remarkably similar to that of healthy controls. The absence of dysbiosis during the earliest stages of disease runs against the hypothesis that *clinical* CD-associated dysbiosis is a causative factor in CD etiology. Rather, the absence of dysbiosis in pre-clinical disease is consistent with a later change due to the different intestinal micro-environment caused by inflammation over time. Clearly, the issue of the microbiome involvement in IBD etiology might be more complex than an all-or-nothing, cause-or-consequence scenarios. The microbiome could still play a role in pathogenesis later in the disease course. For example, an initial alteration in tissue integrity could lead to focal areas of inflammation which might in turn select a dysbiotic bacterial community which can then amplify and perpetuate the disease [113]. Hence, dysbiosis could be a consequence rather than a cause of inflammation—but at the same time it could still be a major driver of the inflammatory process. Further studies are needed to test this and other hypotheses. Regardless, the microbiome dilemma is an example of the potentially vast impact that research focused on preclinical disease could have on our understanding of IBD etiology and pathogenesis.

## 6. The Future

There is no doubt—in the mind of the authors—that pre-clinical IBD will become one of the top clinical and research priorities of the field in the future. As it has already happened with a number of other diseases, knowledge of the natural history of IBD and being able to diagnose it at the pre-clinical stage will greatly impact on its clinical management and it might give us an opportunity to slow down or block disease evolution—or even to find its cause. How can the future of pre-clinical IBD research be envisioned? Large epidemiology studies will continue to be very important and will confirm that environmental factors are crucial in disease development [69,70] and will provide important information on modifiable risk factors [114] such as diet [115] vitamin intake, smoking, oral contraceptives, antibiotics, NSAID’s, physical exercise [114,116] but also childhood hygiene, breastfeeding, air pollution [117]. Clearly, the benefits of lifestyle and environmental changes will be acquired only in the very long term. However the knowledge derived by epidemiology studies could be coupled to genetic tests, disease markers and disease prediction strategies to target specific populations at risk of having/developing IBD (Figure 5). For the purpose, there will be no substitute in the foreseeble future to traditional screening programs based on accurate imaging or pathology. This combined approach might lead to high screening yields and to the identification of patient populations with biologic onset-IBD. These populations could be subjected to observation to characterize the disease natural history, and to interventional RCT’s aimed at blocking disease evolution over time and preventing its symptomatic onset and complications—as it has been done for a long time for other chronic disease (e.g., diabetes) [118]. Subsequent crucial issues will be the benefit to risk ratio of therapeutic interventions, its costs and the implementaton of large scale screening programs. However, the wealth of acquired scientific information in pre-clinical IBD might open the horizon to a complete cure of these life-long, debilitating and expensive diseases.
